# An abnormal screening mammogram causes more anxiety than a palpable lump in benign breast disease

**DOI:** 10.1007/s10549-012-2025-5

**Published:** 2012-03-21

**Authors:** C. M. G. Keyzer-Dekker, L. van Esch, J. de Vries, M. F. Ernst, G. A. P. Nieuwenhuijzen, J. A. Roukema, A. F. W. van der Steeg

**Affiliations:** 1 Department of Pediatric Surgery, Pediatric Surgical Center Amsterdam, Emma Children’s Hospital AMC and VU University Medical Center, PO Box 22660, 1100 DD Amsterdam, The Netherlands; 2 Department of Pediatric Surgery, Beatrix Children’s Hospital, University Medical Center Groningen, Groningen, The Netherlands; 3 Center of Research on Psychology in Somatic Disease (CoRPS), Tilburg University, Tilburg, The Netherlands; 4 Department of Medical Psychology, St. Elisabeth Hospital, Tilburg, The Netherlands; 5 Department of Surgery, Jeroen Bosch Hospital, ‘s Hertogenbosch, The Netherlands; 6 Department of Surgery, Catharina Hospital, Eindhoven, The Netherlands; 7 Department of Surgery, St. Elisabeth Hospital, Tilburg, The Netherlands

**Keywords:** False-positive, Screening mammogram, Anxiety, Depressive symptoms, Quality of life, Benign breast disease

## Abstract

Being recalled for further diagnostic procedures after an abnormal screening mammogram (ASM) can evoke a high state anxiety with lowered quality of life (QoL). We examined whether these adverse psychological consequences are found in all women with benign breast disease (BBD) or are particular to women referred after ASM. In addition, the influence of the anxiety as a personality characteristic (trait anxiety) was studied. Between September 2002 and February 2010 we performed a prospective longitudinal study in six Dutch hospitals. Women referred after ASM or with a palpable lump in the breast (PL), who were subsequently diagnosed with BBD, were included. Before diagnosis (at referral) and during follow-up, questionnaires were completed examining trait anxiety (at referral), state anxiety*,* depressive symptoms (at referral, one, three and 6 months after diagnosis), and QoL (at referral and 12 months). Women referred after ASM (*N* = 363) were compared with women with PL (*N* = 401). A similar state anxiety score was found in both groups, but a lower psychological QoL score at 12 months was seen in the ASM group. In women with not-high trait anxiety those in the ASM group were more anxious with more depressive symptoms at referral, and reported impaired psychological QoL at referral and at 12 months compared with the PL group. No differences were found between ASM and PL in women with high trait anxiety, but this group scored unfavorably on anxiety, depressive symptoms and QoL compared with women with not-high trait anxiety. ASM evokes more anxiety and depressive symptoms and lowered QoL compared with women referred with PL, especially in women who are not prone to anxiety. Women should be fully informed properly about the risks and benefits of breast cancer screening programs. We recommend identifying women at risk of reduced QoL using a psychometric test.

## Introduction

Breast cancer (BC) is the most common cancer among women. In the western world, one in eight women is at risk of developing BC [1, 2]. However, the majority of women visiting the surgical outpatient clinic with breast problems, such as a palpable lump (PL) or an abnormal screening mammogram (ASM), are diagnosed with benign breast disease (BBD) [3]. During the investigation of breast symptoms, women experience increased anxiety and distress [4–6]. Even after a diagnosis of BBD is made, these symptoms persist in a proportion of women [4]. Women with BBD diagnosed after an ASM report ongoing anxiety [7–9] with lowered quality of life (QoL) [10, 11]. In chronically anxious women (i.e., with high trait anxiety), these psychological effects are heightened [6, 10, 12]. Trait anxiety refers to relatively stable individual differences in anxiety proneness [13].

Thus, it is important to evaluate women diagnosed with BBD as the lack of reassurance after the diagnostic work-up and adverse psychological consequences may result in lowered QoL. Although these effects on women with BBD have been previously studied, a comparison between women referred after an ASM or with PL has not been performed before. This comparison is important in the context of the ongoing discussions on whether the advantages of a BC screening program still outweigh the disadvantages (such as the false-positive findings) [14, 15]. Therefore, we examined whether all women with BBD (ASM and PL) experience similar levels of anxiety (state anxiety), depressive symptoms, and changes in QoL during and in the year following the diagnostic work-up. Women attending breast screening usually have no palpable lump in the breast and so are not expecting an ASM. We hypothesized that these women are more alarmed by being recalled for further diagnostic procedures and experience more adverse psychological effects compared with women with a PL. Based on previous studies, we also analyzed the influence of the personality characteristic trait anxiety [10, 16].

In this prospective, longitudinal study comparing ASM with PL, women completed the first set of questionnaires before any diagnostic procedures were performed (at referral).

## Patients and methods

### Participants

Women referred after an ASM or with a PL were eligible for participating in this study. The study was conducted between September 2002 and February 2010 in six Dutch hospitals. The Medical Ethical Committee of the primary research hospital, i.e., St. Elisabeth Hospital, Tilburg, approved the study protocol. This study was part of a larger study analyzing the impact of personality and QoL on morbidity, mortality, and health care consumption in breast disease. Women with recurrent BBD or BC, inability to read and write in Dutch, or (previous) psychiatric illness were excluded. When women were invited to participate in the study and completed the first set of questionnaires, the diagnosis was unknown. All participants gave written informed consent.

Since 1990, BC screening is offered every 2 years to women in the age between 50 and 75 years in the Netherlands. Every year one million women receive an invitation for BC screening mammogram. The overall attendance rate is 80 % [17]. Two-view mammography was used at initial BC screening. All women with an ASM were referred to a dedicated outpatient breast clinic.

### Questionnaires

Questionnaires were completed at referral (before diagnosis was known), and one, three, six, and 12 months after diagnosis. The questionnaires assessed personality at referral (STAI-trait), experienced momentary anxiety (STAI-state) and depressive symptoms (CES-D) at referral until 6 months, and QoL (WHOQOL-bref) at referral and 12 months after diagnosis.

The state-and-trait-anxiety-inventory (STAI) measures two types of anxiety: trait and state. Trait anxiety refers to the tendency to respond to situations perceived as threatening with a rise in anxiety intensity. State anxiety refers to the amount of stress being experienced at the specific moment the measurement is made [13, 18]. In this study, the short 6-item state version and 10-item trait version of the STAI were used [19, 20]. High trait anxiety (HTA) was defined as a score greater than 22. The reliability and validity of the short versions are considered good [19, 20].

The Center for Epidemiological Studies-depression scale (CES-D) was used to assess depressive symptoms. It measures both the presence and the degree of depressive symptoms. The psychometric properties are good [21, 22].

The World Health Organization Quality of Life assessment instrument-Bref (WHOQOL-Bref) is a short version of the WHOQOL-100 [23, 24]. The WHOQOL-Bref consists of questions assessing QoL within four domains (physical health, psychological health, social relationships, and environment) and a general evaluative facet (overall QoL and general health). The psychometric properties of the WHOQOL-Bref have been demonstrated to be good in women with benign breast disease [3].

Women were also asked to complete a questionnaire concerning demographic characteristics. The medical data concerning patient and mammography characteristics were obtained from the medical records.

### Statistics

Women who did not complete all questionnaires during follow up, were excluded from further analysis and considered as drop-outs. Chi-square tests and independent *t* tests were used to compare women in the non drop-out and drop-out groups, and in the ASM or PL groups with regard to demographic (age, children, marital status, paid work, and educational level) and personality (trait anxiety) characteristics at baseline. Differences in demographic characteristics were used as covariates in the subsequent analysis.

A repeated measures general linear model was used to examine scores on state anxiety and depressive symptoms (at referral until 6 months), and QoL (at referral and 12 months) across time (i) in the two groups ASM or PL, and (ii) in women with HTA or not-high score on trait anxiety (NHTA) in ASM or PL group. A *P* value <0.05 was considered statistical significant. All analyses were performed with the Statistical Package for Social Sciences (SPSS version 18.0).

## Results

During the study period, 1145 women were diagnosed with BBD. During follow-up, 381 women did not complete all questionnaires, and were excluded from further analysis. Women in the drop-out group were less educated (*P* = 0.016) and scored higher on trait anxiety (*P* < 0.001) compared with the group that remained in the study. There was no difference concerning referral after ASM or PL.

In total, 764 women were analyzed at referral, 363 women in the ASM group, and 401 in the PL group. At referral, significant differences were observed concerning demographics between the two groups (Table 1). Women in the ASM group were older (*P* < 0.001), more often had children (*P* = 0.009), and less often had paid work (*P* < 0.001). There was no difference between the two groups concerning trait anxiety at referral.

**Table 1:** Demographic and psychological characteristics comparing two groups: women with benign breast disease referred with an abnormal screening mammogram (ASM) or with a palpable lump in breast (PL)

	ASM (*n* = 363)	PL (*n* = 401)	*P* value
Demographics			
Mean age (SD)	56.2 (6.8)	46.5 (10.9)	**<0.001**
Partner *n* (%)	308 (85)	348 (87)	0.315
Children *n* (%)	315 (88)	322 (81)	**0.009**
Education low/moderate *n* (%)	290 (80)	306 (76)	0.053
Paid work *n* (%)	194 (54)	289 (72)	**<0.001**
Personality			
High score on trait anxiety *n* (%)	75 (21)	85 (21)	0.856
Psychological factors mean scores (SD)			
State anxiety at referral	12.9 (4.0)	12.4 (3.8)	0.074
State anxiety 6 months	10.4 (3.4)^a^	10.4 (3.5)^a^	0.988
Depressive symptoms at referral	8.8 (8.2)	7.2 (7.6)	**0.007**
Depressive symptoms 6 months	6.5 (7.0)^a^	6.0 (7.1)^a^	0.407
General quality of life at referral	7.9 (1.4)	7.9 (1.4)	0.732
General quality of life 12 months	7.7 (1.4)	8.0 (1.4)	0.385

*SD* standard deviation. *P* value <0.05 considered significant and presented in *bold*

^a^Scores diminished significantly at 6 months compared with scores at referral

At referral, the mean scores for state anxiety were comparable in the ASM and PL groups (*P* = 0.074; Table 1). In both groups, the state anxiety scores significantly decreased after 1 month compared with the scores at referral (*P* < 0.001; Table 1). Concerning depressive symptoms, a higher mean score was found in ASM compared with PL at referral (*P* = 0.007), in both groups scores significantly decreased at 1 month compared with the scores at referral (*P* < 0.001; Table 1). After 1 month, no differences between the two groups were found. From 1 month follow-up scores on state anxiety and depressive symptoms remained similar until 6 months in both groups (Table 1). Concerning the scores on QoL, there were no differences between the ASM and the PL groups at referral. At 12 months, women in the ASM group scored lower on psychological QoL (*P* = 0.022) compared with the PL group.

In the subanalysis, women were divided in four groups based on referral after ASM or with PL and their scores on trait anxiety (HTA or NHTA).

### High trait anxiety

**Fig. 1:**
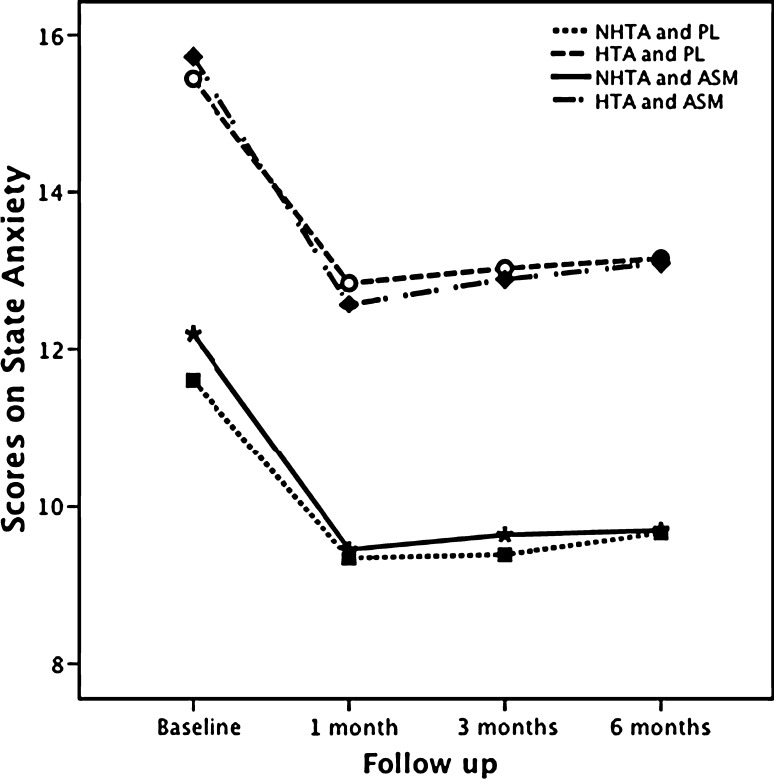
State anxiety for women with high or not-high trait anxiety comparing ASM and PL groups at referral and during follow-up until 6 months. *HTA* high trait anxiety, *PL* palpable lump in the breast, *ASM* abnormal screening mammogram, and *NHTA* not-high trait anxiety

**Fig. 2:**
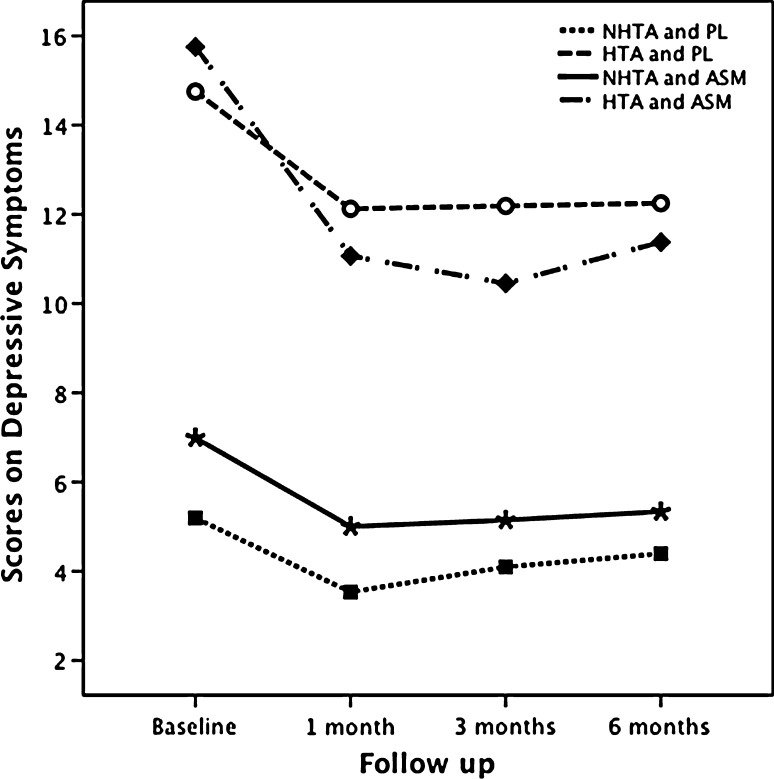
Depressive symptoms for women with high or not-high trait anxiety comparing ASM and PL groups at referral and during follow-up until 6 months. *HTA* high trait anxiety, *PL* palpable lump in the breast, *ASM* abnormal screening mammogram, and *NHTA* not-high trait anxiety

Women with HTA (*N* = 160) scored higher at all measurement moments on state anxiety, depressive symptoms, and lower on QoL compared with women with NHTA (*P* < 0.001; Table 2). Within the group with HTA, women in the PL and ASM groups scored similar on state anxiety, depressive symptoms, and QoL (Table 2; Figs. 1, 2). In both groups, scores on depressive symptoms decreased significantly at 1 month compared with scores at referral (*P* < 0.001). The only difference was a higher score on psychological QoL at referral in the ASM group (*P* = 0.029).

**Table 2:** Scores at referral and during follow up on state anxiety, depressive symptoms, and general quality of life (QoL) comparing four groups based on referral after abnormal screening program (ASM) or with palpable lump in breast (PL) and high trait anxiety (HTA) or not-high trait anxiety (NHTA)

Mean scores (SD)	ASM (*n* = 75)		PL (*n* = 85)		*P* value
HTA^a^	At referral	6 months	At referral	6 months	
State anxiety	15.7 (3.4)	13.1 (3.6)	15.5 (3.5)	13.2 (3.6)	NS
Depressive symptoms	15.8 (9.3)	11.4 (9.3)^b^	14.7 (9.6)	12.3 (8.8)^b^	NS

	At referral	12 months	At referral	12 months	
General QoL	6.9 (1.4)	6.9 (1.6)	6.7 (1.5)	6.7 (1.3)	NS

	ASM *n* = 288		PL *n* = 316		*P* value
NHTA^a^	At referral	6 months	At referral	6 months	
State anxiety	12.2 (3.9)	9.7 (3.0)^b^	11.6 (3.4)	9.7 (3.1)^b^	*R* 0.047
Depressive symptoms	7.0 (6.8)	5.3 (5.6)	5.2 (5.4)	4.4 (5.4)	*R* < 0.001

	At referral	12 months	At referral	12 months	
General QoL	8.1 (1.4)	8.0 (1.3)	8.2 (1.2)	8.3 (1.2)	NS

*P* value <0.05 considered significant. *SD* standard deviation, *NS* not significant, *R* significant difference at referral comparing ASM and PL

^a^All values in the HTA groups are significant different compared with NHTA groups (*P* < 0.001)

^b^Scores significantly changed at 6 months compared with scores at referral

### Not-high trait anxiety

In women with NHTA (*N* = 604), higher scores on state anxiety at referral were found in the ASM group compared with the PL group (*P* = 0.047). During follow-up, these scores significantly diminished after 1 month compared with the scores at referral in both groups (*P* < 0.001; Table 2; Fig. 1), and the scores remained similar until 6 months compared with 1 month, without differences between groups. Scores on depressive symptoms were higher in ASM at referral compared with PL (*P* < 0.001; Table 2; Fig. 2). In both groups, scores after 1 month remained similar during follow-up. Concerning QoL, women in the ASM group scored lower on psychological QoL at referral (*P* = 0.022) and at 12 months (*P* = 0.005) compared with the PL group.

## Discussion

The discussion concerning the disadvantages of the BC screening program, such as false-positive findings, is still ongoing and contributing to the screening controversy [14, 15]. The adverse psychological consequences after a false-positive screening mammogram are already described before [7–11, 16]. However, to our knowledge, a comparison between women with BBD referred after ASM or PL has not yet been performed. We hypothesized that women referred after ASM experience more adverse psychological effects compared with women referred with PL.

As previously found, the negative effects in our study were strengthened by the personality characteristic trait anxiety, i.e., women with HTA scored unfavorably on state anxiety, depressive symptoms, and QoL, compared with women not prone to anxiety [6, 10, 12]. Before diagnosis was known, all women scored higher on state anxiety and depressive symptoms compared with 1 month after diagnosis, when women were relieved that BC was not found. In addition, we have found that within women not prone to anxiety, those in the ASM group were more anxious before diagnosis was known and experienced more depressive symptoms at referral compared with all women with PL. In addition, those women reported impaired psychological QoL at referral and 1 year after diagnosis compared with PL. In chronically anxious women higher scores on depressive symptoms at referral were found compared with 1 month after diagnosis, regardless of being referred after an ASM or with a PL.

Thus, the negative impact of a false-positive screening mammogram on anxiety, depressive symptoms, and QoL is especially found in women who do not have a high propensity for anxiety, confirming our previous findings [16]. These effects cannot be considered as a normal response to the diagnostic work up for breast disease, because not every woman responds similar to the threat of possibly having BC. The fact that women not prone to anxiety are affected more implies that being recalled for further diagnostic procedures after an ASM is a serious psychological problem, especially because the adverse effects persist at least 1 year after the diagnostic process showed by the lowered QoL.

The present findings contribute to the ongoing screening controversy: are the advantages of the BC screening program still in balance with the disadvantages? Recent data has suggested that screening has little detectable impact on BC mortality [25]. In addition, several publications have discussed the benefits and harms of the BC screening program [14, 15, 26–31]. Currently the decision to participate in the BC screening program is based upon information in favor of screening. The risk for possible adverse psychological consequences, overdiagnosis and overtreatment are not mentioned in the provided information [14, 28–30, 32, 33].

## Conclusions

This study reveals that women recalled after an ASM experience higher state anxiety and depressive symptoms at referral with lowered QoL 1 year after diagnosis, compared with women with a PL, especially women not prone to anxiety. Therefore, we recommend that women should be informed properly concerning the benefits and risks of the BC screening program, in particular mentioning the adverse psychological consequences after a false-positive screening mammogram. In addition, at intake women should be offered a psychometric test to identify those who are at risk for impaired QoL.

## Ethical standards

The study was approved by the Medical Ethical Committee.

## Abbreviations

BC: Breast cancer
PL: Palpable lump in the breast
ASM: Abnormal screening mammogram
BBD: Benign breast disease
QoL: Quality of life
HTA: High trait anxiety
NHTA: Not-high trait anxiety
